# Unraveling the Factors Determining Development of Type 2 Diabetes in Women With a History of Gestational Diabetes Mellitus Through Machine-Learning Techniques

**DOI:** 10.3389/fphys.2022.789219

**Published:** 2022-02-17

**Authors:** Ludovica Ilari, Agnese Piersanti, Christian Göbl, Laura Burattini, Alexandra Kautzky-Willer, Andrea Tura, Micaela Morettini

**Affiliations:** ^1^Department of Information Engineering, Università Politecnica delle Marche, Ancona, Italy; ^2^Department of Obstetrics and Gynecology, Medical University of Vienna, Vienna, Austria; ^3^Division of Endocrinology and Metabolism, Department of Internal Medicine III, Medical University of Vienna, Vienna, Austria; ^4^Metabolic Unit, CNR Institute of Neuroscience, Padua, Italy

**Keywords:** pathophysiology, predictive biomarker, disease prediction, statistical learning, logistic regression, mathematical model

## Abstract

Gestational diabetes mellitus (GDM) is a type of diabetes that usually resolves at the end of the pregnancy but exposes to a higher risk of developing type 2 diabetes mellitus (T2DM). This study aimed to unravel the factors, among those that quantify specific metabolic processes, which determine progression to T2DM by using machine-learning techniques. Classification of women who did progress to T2DM (labeled as PROG, *n* = 19) vs. those who did not (labeled as NON-PROG, *n* = 59) progress to T2DM has been performed by using Orange software through a data analysis procedure on a generated data set including anthropometric data and a total of 34 features, extracted through mathematical modeling/methods procedures. Feature selection has been performed through decision tree algorithm and then Naïve Bayes and penalized (L2) logistic regression were used to evaluate the ability of the selected features to solve the classification problem. Performance has been evaluated in terms of area under the operating receiver characteristics (AUC), classification accuracy (CA), precision, sensitivity, specificity, and F1. Feature selection provided six features, and based on them, classification was performed as follows: AUC of 0.795, 0.831, and 0.884; CA of 0.827, 0.813, and 0.840; precision of 0.830, 0.854, and 0.834; sensitivity of 0.827, 0.813, and 0.840; specificity of 0.700, 0.821, and 0.662; and F1 of 0.828, 0.824, and 0.836 for tree algorithm, Naïve Bayes, and penalized logistic regression, respectively. Fasting glucose, age, and body mass index together with features describing insulin action and secretion may predict the development of T2DM in women with a history of GDM.

## Introduction

Diabetes is a chronic metabolic disease characterized by the presence of high levels of glucose in the blood (i.e., hyperglycemia). Several pathogenic processes can be at the basis of diabetes development leading to the identification of different diabetes categories, namely, type 1 diabetes mellitus (T1DM), type 2 diabetes mellitus (T2DM), and gestational diabetes mellitus (GDM; American Diabetes Association, 2020). Among these, T2DM is the most common, and the three main processes underlying its development are tissue resistance to the action of insulin (i.e., insulin resistance), altered insulin secretion by the pancreas, and altered insulin clearance (i.e., removal of insulin from the blood in the entire organism) (Bizzotto et al., 2021). According to the most recent definition, GDM is defined as a diabetes diagnosed in the second or third trimester of pregnancy that was not clearly overt diabetes prior to gestation (American Diabetes Association, 2020); although it usually resolves at the end of the pregnancy, women who experienced GDM are known to have a higher risk of developing T2DM later in their life (American Diabetes Association, 2020). Therefore, determination of factors influencing the development of T2DM may also shed light on GDM and potentially accelerate opportunities for prevention and treatment (Plows et al., 2018).

In the past years, research on T2DM has been taking advantage on one side of the availability of a huge amount of heterogeneous data and on the other side of machine-learning techniques that can be used to automatically extract knowledge from them (Kavakiotis et al., 2017). The application of machine-learning techniques to this field has been done on a wide variety of data and has been aimed at different purposes, for example, early diagnosis (Perveen et al., 2016; Zheng and Zhang, 2017; El_Jerjawi and Abu-Naser, 2018; Sarwar et al., 2018; Zou et al., 2018; Bernardini et al., 2020; Garcia-Carretero et al., 2021), estimation of T2DM risk (Dalakleidi et al., 2017; Talaei-Khoei and Wilson, 2018; Garcia-Carretero et al., 2020), detection of subjects in the general population affected by T2DM or prediabetes (Yu et al., 2010), T2DM characterization and classification (Maniruzzaman et al., 2017; Bernardini et al., 2019), and T2DM care (Huang et al., 2007).

However, the application of such techniques to determine factors influencing the development of T2DM in women with a history of GDM has still been scarcely explored, and few studies addressing this topic have focused on metabolomics and/or lipidomics (Lappas et al., 2015; Allalou et al., 2016; Khan et al., 2019), which consist of the identification and determination of the set of metabolites or specific metabolites, such as lipids, in biological samples (i.e., tissues, cells, fluids, or organisms) under normal conditions in comparison with altered states promoted by disease or specific stimuli (e.g., drug treatment, dietary/activity regimen, or environmental modulation) (Klassen et al., 2017). Although metabolomics and lipidomics are promising approaches to allow a more personalized control of T2DM, there are still many limitations and challenges that need to be addressed for the translation of the research outcomes into clinical tests (Pinu et al., 2019).

A more traditional approach with respect to metabolomics and lipidomics in the field of T2DM research consists of the extraction, sometimes with sophisticated mathematical modeling methodologies, of features describing parameters of physiological interest from raw data measured during standard clinical tests (Mari et al., 2020). However, to the best of our knowledge, the application of machine-learning techniques to analyze data set containing this kind of features has never been performed in women with a history of GDM and at risk of developing T2DM. Thus, the aim of this study was to unravel the factors, among those that quantify specific metabolic processes, which determine the development of T2DM in women with a history of GDM by using machine-learning techniques. The rest of the article is structured as follows: the section “Materials and Methods” presents the clinical data that were used, how the features have been extracted from them to generate the data set to be analyzed, and how the classification problem was performed; the sections “Results” and “Discussion” present the obtained results and discuss them, respectively; the final section “Conclusion” concludes the presentation.

## Materials and Methods

### Clinical Data

Data used in this study were already analyzed in previous studies (Tura et al., 2012, 2020) and were collected in agreement with the Declaration of Helsinki and upon approval of the local Ethics Committee. Written informed consent for participation in the study has been given by each participant. A group of 78 women who experienced a history of GDM were considered. All women were analyzed early postpartum (4–6 months after delivery) and then re-examined over a period of up to 7 years. During the follow-up period, some women developed T2DM (*n* = 19), whereas the others did not develop T2DM (*n* = 59). All women were non-diabetic at the time of the first analysis (early postpartum), and none of the women was treated with antidiabetic agents before the possible onset of T2DM.

All women underwent a frequently sampled insulin modified intravenous glucose tolerance test (IM-IVGTT) early postpartum and at the end of the follow-up period. Glucose was injected at time 0–0.5 min (300 mg/kg), and insulin (0.03 IU/kg) was infused intravenously at time 20 min for 5 min. Venous blood samples were collected at fasting and for 180 min following glucose injection (at 3, 4, 5, 6, 8, 10, 14, 19, 22, 27, 30, 35, 40, 50, 70, 100, 140, and 180 min) for the measurement of glucose (mmol⋅L^–1^), insulin (pmol⋅L^–1^), and C-peptide (pmol⋅L^–1^) plasma concentrations.

### Feature Extraction and Data Set Generation

From the original clinical data, a new data set has been generated by including for each woman her anthropometric data [i.e., age, body weight (BW), height (h), and body mass index (BMI)] and extracted features, related to parameters of physiological interest, derived by applying mathematical models and methods to her IM-IVGTT data at early postpartum condition. In the generated data set, women who progressed to T2DM have been labeled as progressors (PROG), whereas those who did not progress have been labeled as non-progressors (NON-PROG). In detail, extracted features included the following: (i) mean areas under the glucose, insulin, and C-peptide curves (*G*_MEAN_, *I*_MEAN_, and Cp_MEAN_, respectively), computed as the areas under the curve (AUCs) divided by the test duration (Morettini et al., 2020b); (ii) area under the insulin curve during the first phase (AUC_INS–1P_) and second phase (AUC_INS–2P_) of the test; (iii) rate of glucose disappearance before and after insulin infusion (*K*_G1_ and *K*_G2_, respectively), computed as the slope (absolute value) of log_e_ glucose multiplied by 100 in the 10–20- and 20–40-min intervals, respectively (Morettini et al., 2020a); (iv) insulin-dependent and insulin-independent glucose disappearance, quantified through insulin sensitivity (*S*_I_) and glucose effectiveness (*S*_G_), respectively, as assessed by the minimal model of glucose kinetics, which also provides an estimate of glucose distribution volume (*V*) (Pacini et al., 1998); (v) basal insulin effect (BIE) and glucose effectiveness at zero insulin (GEZI), representing *S*_G_ components (Kahn et al., 1990); (vi) first-phase insulin secretion, quantified through the acute insulin response (AIR; Johnston et al., 1987) and the acute C-peptide response (ACPR) indexes, which have been computed as the mean of suprabasal insulin and C-peptide curves, respectively, in the time interval 3–8 min during the IM-IVGTT; and (vii) combined contribution of insulin action and β-cell function, assessed through the disposition index (DI), computed as the product between *S*_I_ and AIR (Kahn et al., 1993).

Moreover, insulin secretion rate [ISR(t)] has been derived by deconvolution of plasma C-peptide concentrations during the IM-IVGTT (Van Cauter et al., 1992), and exploiting it, the following features were extracted: (i) basal secretion rate (BSR) and β-cell responsivity to glucose (Φ_1c_); (ii) AUC of ISR(t) for the whole test (AUC_SECR_) and also for the first part (AUC_SECR–1P_) and the second part (AUC_SECR–2P_) of the test; (iii) insulin clearance during the whole test (CL_MEAN_) and also during the first part (CL_MEAN–1P_) and second part (CL_MEAN–2P_) of the test (Morettini et al., 2020b); and (iv) insulin clearance with segregation of its hepatic (FE_L_) and extra-hepatic (CL_P_) contribution, derived by applying the approach proposed by Polidori et al. (2016).

Peak insulin after glucose injection (*I*_PEAK–FIRST_) and after insulin injection (*I*_PEAK–INJECT_), peak C-peptide (*C*_PEAK_), and glucose dose injected (DOSE) have been also included in the generated data set. Fasting plasma glucose (g_b_) has also been included since it is the most important clinical marker for the diagnosis of glucose tolerance deterioration (American Diabetes Association, 2020). Description of the generated data set is summarized in Table 1.

**TABLE 1:** Description of all the features included in the generated data set.

Name	Acronym	Units
Age	age	years
Body weight	BW	kg
Height	h	cm
Body mass index	BMI	kg⋅m^−2^
Basal glucose	g_b_	mg⋅dL^−1^
Mean area under the glucose curve	G_MEAN_	mmol⋅L^−1^
Mean area under the insulin curve	I_MEAN_	pmol⋅L^−1^
Mean area under the C-peptide curve	Cp_MEAN_	pmol⋅L^−1^
Area under the insulin curve during the 1st phase of test	AUC_INS–1P_	pmol⋅L^−1^⋅min^–1^
Area under the insulin curve during the 2nd phase of test	AUC_INS–2P_	pmol⋅L^−1^⋅min^–1^
Disappearance rate of glucose before insulin injection	K_G1_	%/min
Disappearance rate of glucose after insulin injection	K_G2_	%/min
Insulin sensitivity	S_I_	10^−4^⋅min^−1^/(μU⋅mL^−1^)
Glucose effectiveness	S_G_	min^–1^
Distribution volume of glucose	V	L
Basal insulin effect of glucose effectiveness	BIE	10^–3^⋅min^–1^
Glucose effectiveness at zero insulin	GEZI	10^–2^⋅min^–1^
Mean of suprabasal insulin in the time interval 3–8 min	AIR	pmol⋅L^−1^
Mean of suprabasal C-peptide in the time interval 3–8 min	ACPR	pmol⋅L^−1^
Disposition index	DI	min^–1^
Basal secretion rate	BSR	pmol⋅L^−1^⋅min^−1^
β-cell responsivity to glucose	Φ_1c_	(pmol⋅L^−1^⋅min^−1^)/(mg⋅dL^−1^)
Area under the secretion curve during the entire test	AUC_SECR_	pmol
Area under the secretion curve during the 1st phase of test	AUC_SECR–1P_	pmol
Area under the secretion curve during the 2nd phase of test	AUC_SECR–2P_	pmol
Mean insulin clearance during the entire test	CL_MEAN_	L⋅min^−1^
Mean insulin clearance during the 1st phase of test	CL_MEAN–1P_	L⋅min^−1^
Mean insulin clearance during the 2nd phase of test	CL_MEAN–2P_	L⋅min^−1^
Extra-hepatic insulin clearance	CL_P_	L⋅min^−1^
Hepatic insulin clearance	FE_L_	%
Peak insulin after glucose injection	I_PEAK–FIRST_	μU⋅mL^−1^
Peak insulin after insulin injection	I_PEAK–INJECT_	μU⋅mL^−1^
Peak C-peptide	C_PEAK_	ng⋅dL^−1^
Glucose dose injected	DOSE	g

### Classification Problem and Data Analysis

The classification problem consisted of classifying PROG vs. NON-PROG considering the complete generated data set as input. Data analysis has been performed by using Orange (version 3.28)^1^, an open-source data visualization, machine learning, and data mining toolkit which provides a visual programming front-end (called Orange Canvas) for explorative rapid qualitative data analysis and interactive data visualization (Demšar et al., 2013). The input data set has been preprocessed by detecting the outliers in PROG and NON-PROG through local outlier factor (LOF), which measures the local deviation of the density of a given sample with respect to its neighbors (Breuniq et al., 2000), and then parameters have been set as follows: “Contamination” = 4%, “Neighbors” = 20, and “Metric” = Euclidean. Cases (PROG/NON-PROG) identified as outliers by LOF were removed from the data set. The preprocessed data set has been given as input to a classification algorithm based on decision tree, which determines the best predictive features by splitting the data into nodes by class purity; information gain ratio has been used as a score to evaluate features for splitting instances in a node. Parameters of the decision tree were set as follows: “Minimum number of instances in leaves” = 3, meaning that the algorithm does not construct a split that would put less than 3 training examples into any of the branches; “Do not split subsets smaller than” = 5, which forbids the algorithm to split the nodes with less than 5 instances; “Maximal tree depth” = 100, which limits the depth of the tree to 100 node levels; “Induce binary tree,” which builds a binary tree; and “Stop when majority reaches 95%,” indicating that the algorithm stops splitting the nodes after 95% of the classified example is reached.

**FIGURE 1:**
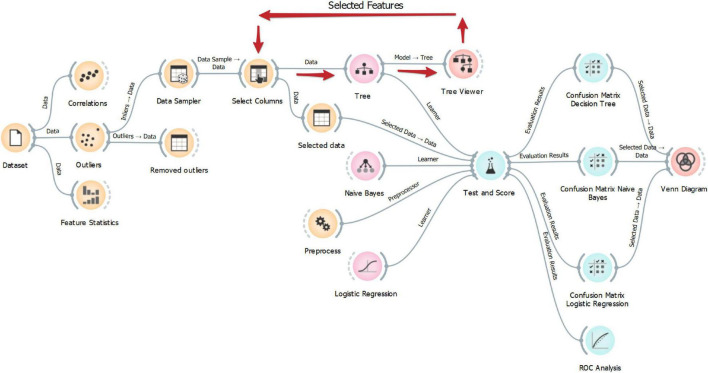
Orange workflow for data analysis.

To evaluate the ability of the selected features to solve the classification problem, they were given as input to different classification algorithms, namely, Naïve Bayes (a probabilistic classifier based on Bayes’ theorem with the assumption of feature independence) and penalized logistic regression. Naïve Bayes did not require any setting for the parameters, whereas logistic regression parameters were set as follows: “Regularization type” = RIDGE (L2), Cost strength (C) = 1. L2 regularization has been used since penalized regression models showed advantages in scenarios with small sample size and multiple highly correlated metabolic variables in previous studies (Göbl et al., 2015). All the classification algorithms (i.e., decision tree, Naïve Bayes, and penalized logistic regression) have been built by using a 5-fold cross-validation; features given as input of the classification algorithms have been normalized (“Mean” = 0, “Variance” = 1) to adjust their values to a common scale. The average performance of the classification algorithms on 5-fold has been evaluated. The Orange workflow for data analysis is reported in Figure 1.

### Performance Measures

By considering PROG class as positive and NON-PROG class as negative, the PROG cases classified as PROG by the classification algorithm were considered true positive (TP); the NON-PROG cases classified as NON-PROG by the classification algorithm were considered true negative (TN); the PROG cases classified as NON-PROG by the classification algorithm were considered false negative (FN); and the NON-PROG cases classified as PROG by the classification algorithm were considered false positive (FP).

The performance of each classification algorithm has been evaluated by computing the AUC of the receiver operating characteristics (ROC) and by computing the following measures: classification accuracy (CA, i.e., the proportion of cases correctly identified by the classification algorithm), precision (i.e., the proportion of TP among cases classified as positive), sensitivity (also indicated as recall, i.e., the proportion of positive cases that are correctly classified), specificity (i.e., the proportion of negative cases that are correctly classified), and F1 (i.e., the weighted harmonic mean of precision and recall).

### Statistics and Classification Algorithms Comparison

The Lilliefors test was used to evaluate the hypothesis that each variable had a normal distribution with unspecified mean and variance. Normally distributed variables were presented as mean ± standard deviation (SD); skewed distributed variables were presented as median [interquartile range, IQR]. Differences in mean/median values of variables between the two groups were tested by unpaired Student’s *t*-test for equal mean and equal but unknown variance or Wilcoxon rank-sum test for equal median.

Comparison of classification algorithms in terms of correctly classified and misclassified cases has been performed by using Venn diagrams. Moreover, comparison of the performance measures (i.e., AUC, CA, precision, sensitivity, specificity, and F1) has been performed by using the Bayesian interpretation of the pairwise Student’s *t*-tests (Corani and Benavoli, 2015). The statistical significance level was set at 5% for all the tests.

## Results

**FIGURE 2:**
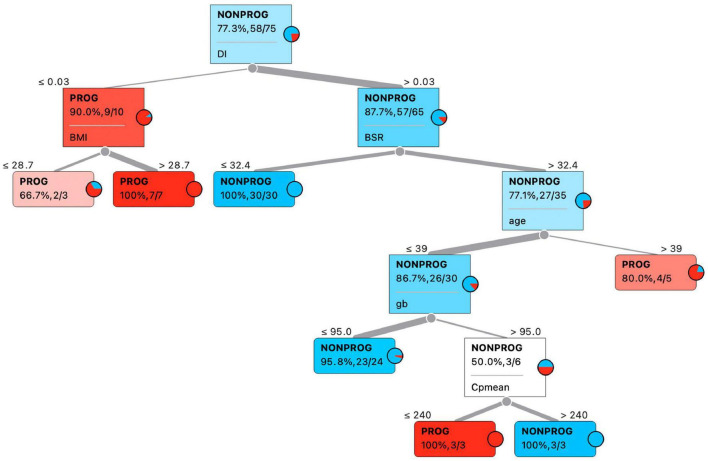
Best predictive features according to decision tree classification algorithm.

Characteristics of the preprocessed data set are reported in Table 2. With respect to the generated data set given as input to the data analysis procedure, three outliers have been removed (1 NON-PROG and 2 PROG), thus resulting in a total of 75 cases; comparing the characteristics of NON-PROG and PROG, 25 out of 34 characteristics have been found statistically different. Feature selection performed through decision tree provided six features for the classification of PROG vs. NON-PROG, specifically DI, BMI, BSR, age, g_b_, and Cp_MEAN_; thresholds identified by decision tree were 0.3 min^–1^, 28.7 kg⋅m^–2^, 32.4 pmol⋅L^−1^⋅min^−1^, 39 years, 95 mg⋅dl^−1^, and 240 pmol⋅L^−1^ for DI, BMI, BSR, age, g_b_, and Cp_MEAN_, respectively (Figure 2). All the selected features have been found to be significantly different between PROG and NON-PROG; however, statistical difference in Cp_MEAN_ is not strongly significant (*p* = 0.04, refer to Table 2).

**TABLE 2:** Characteristics of the preprocessed data set.

Characteristics	NON-PROG (*n* = 58)	PROG (*n* = 17)	*p*-value
age	33.3 ± 4.2	36.6 ± 4.7	**<0.01**
BW	65.8 [15.0]	75.0 [18.8]	**0.03**
h	164 [12]	158 [12]	**0.04**
BMI	25.4 ± 3.9	30.6 ± 6.4	**<0.001**
g_b_	84 [8]	96 [11.50]	**<0.001**
G_MEAN_	5.1 [0.7]	5.9 [0.8]	**<0.001**
I_MEAN_	201.8 [78.3]	211.9 [102.8]	n.s.
Cp_MEAN_	182.4 [97.2]	240.9 [65.4]	**0.04**
AUC_INS–1P_	2125.5 [1053.2]	1520.9 [1120.0]	**<0.01**
AUC_INS–2P_	34468.1 [1417.8]	36992.9 [18872.1]	n.s.
K_G1_	1.93 [0.88]	1.56 [0.63]	**<0.001**
K_G2_	4.7 ± 1.8	3.4 ± 1.8	**0.01**
SI	4.7 [2.7]	3.1 [2.0]	**0.02**
S_G_	0.022 [0.005]	0.018 [0.008]	**<0.01**
V	13.4 [1.36]	13.9 [1.00]	n.s.
BIE	3 [2.4]	2.4 [1.5]	n.s.
GEZI	1.9 [0.7]	1.7 [0.6]	n.s.
AIR	194.4 [126.5]	132.8 [103.0]	**<0.01**
ACPR	254.0 [138.8]	158.2 [150.0]	**<0.01**
DI	1.36 [1.25]	0.52 [0.76]	**<0.001**
BSR	31.8 [10.4]	39.6 [10.9]	**<0.001**
Φ_1c_	67.80 [31.90]	42.02 [33.31]	**<0.01**
AUC_SECR_	24734.9 [15607.7]	32324.0 [11317.6]	**0.03**
AUC_SECR–1P_	7146.2 [3591.8]	6252.9 [3285.6]	**0.02**
AUC_SECR–2P_	17450.7 [15298.7]	27324.0 [9849.6]	**<0.01**
CL_MEAN_	0.69 [0.42]	0.78 [0.47]	**0.03**
CL_MEAN–1P_	3.30 [0.92]	4.17 [1.76]	n.s.
CL_MEAN–2P_	0.53 [0.39]	0.68 [0.35]	**<0.01**
CL_P_	0.39 ± 0.42	0.56 ± 0.53	n.s.
FE_L_	0.53 [0.14]	0.50 [0.13]	n.s.
I_PEAK–FIRST_	53.2 [39.5]	36.0 [39.3]	**<0.01**
I_PEAK–INJECT_	493 [281]	559 [150]	n.s.
C_PEAK_	465 [197]	380 [140]	**0.04**
DOSE	19.7 [4.5]	22.5 [5.6]	**0.02**

*Data are presented as mean ± standard deviation or median [interquartile range]. Significance level: p-values < 0.05. n.s., not significant. Bold values indicate significant differences.*

**FIGURE 3:**
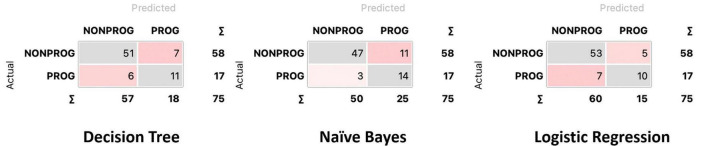
Confusion matrix for decision tree, Naïve Bayes, and logistic regression.

**FIGURE 4:**
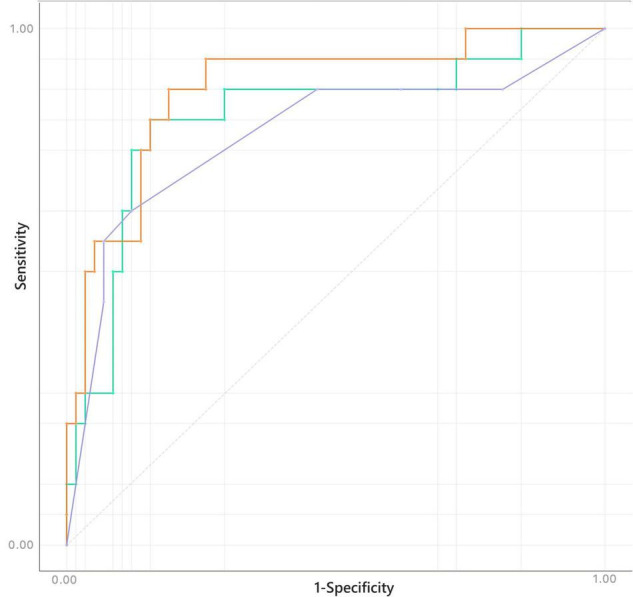
Receiver operating characteristics (ROC) for decision tree (purple), Naïve Bayes (green), and logistic regression (orange).

**FIGURE 5:**
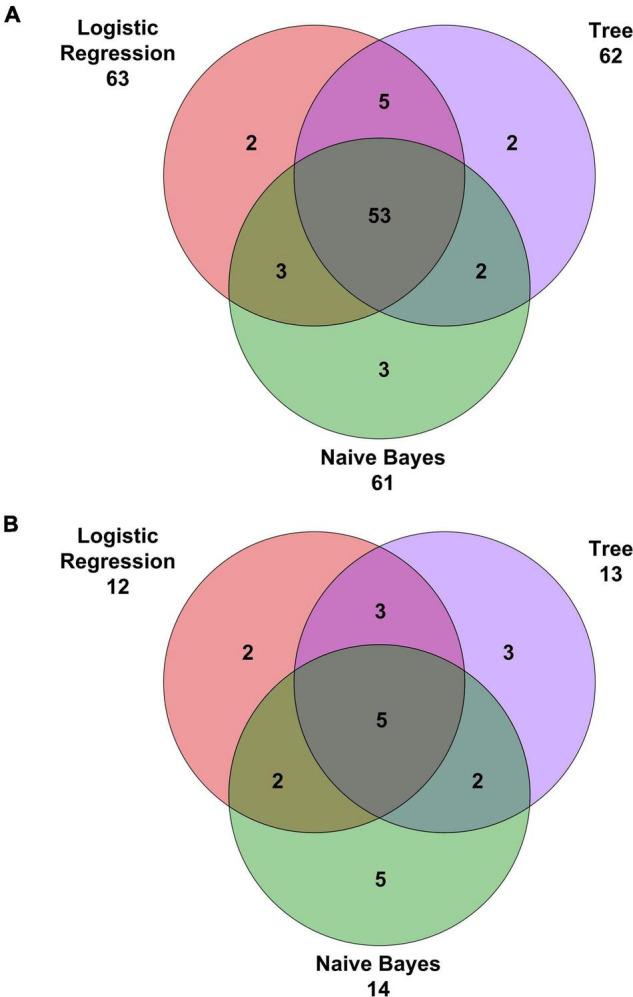
Venn diagrams with **(A)** correct classifications and **(B)** misclassifications.

Confusion matrices for decision tree, Naïve Bayes, and penalized logistic regression are reported in Figure 3. Correctly classified cases by each classification algorithms were 62, 61, and 63, respectively. The related ROC curves are reported in Figure 4. Results related to the performance of each classification algorithm in terms of AUC, CA, precision, sensitivity, specificity, and F1 are reported in Table 3, and comparison among algorithms are reported in Table 4. Penalized logistic regression outperformed tree and Naïve Bayes in terms of AUC (88.7 vs. 71.7%), CA (57.5 vs. 62.0%), sensitivity (57.5 vs. 62.0%), and F1 (52.8 vs. 54.0%), but not in terms of precision and specificity (in which Naïve Bayes was superior with 64.1 and 86.7%, respectively). The Venn diagrams for comparison among models of correctly classified/misclassified cases are shown in Figure 5; correctly classified or misclassified cases by all the classification algorithms were 53 and 5, respectively.

**TABLE 3:** Performance of the three classification algorithms.

Classification algorithm			

Performance measures	Tree	Naïve Bayes	Logistic regression
AUC	0.795	0.831	0.884
CA	0.827	0.813	0.840
Precision	0.830	0.854	0.834
Sensitivity	0.827	0.813	0.840
Specificity	0.700	0.821	0.662
F1	0.828	0.824	0.836

**TABLE 4:** Comparison of the performance measures among classification algorithms through Bayesian interpretation of the pairwise Student’s *t*-tests.

	AUC		
	Tree	Naïve Bayes	Logistic regression
Tree		26.2	11.3
Naïve Bayes	73.8		28.3
Logistic regression	88.7	71.7	

	**CA**		
	Tree	Naïve Bayes	Logistic regression

Tree		57.5	42.5
Naïve Bayes	42.5		38.0
Logistic regression	57.5	62.0	

	**Precision**		
	Tree	Naïve Bayes	Logistic regression

Tree		35.9	42.0
Naïve Bayes	64.1		54.0
Logistic regression	58.0	46.0	

	**Sensitivity**		
	Tree	Naïve Bayes	Logistic regression

Tree		57.5	42.5
Naïve Bayes	42.5		38.0
Logistic regression	57.5	62.0	

	**Specificity**		
	Tree	Naïve Bayes	Logistic regression

Tree		13.3	57.6
Naïve Bayes	86.7		86.6
Logistic regression	42.4	13.4	

	**F1**		
	Tree	Naïve Bayes	Logistic regression

Tree		52.0	47.2
Naïve Bayes	48.0		46.0
Logistic regression	52.8	54.0	

*Probability that the score for the classification algorithm in the row is higher than that of the classification algorithm in the column is reported.*

## Discussion

This study provided an overview of the most relevant factors that may determine the development of T2DM in women with a history of GDM. Machine-learning techniques were applied to a data set appropriately generated by including features quantifying specific metabolic processes such as insulin sensitivity, β-cell function, and insulin clearance, which are all relevant processes underlying T2DM development (Bizzotto et al., 2021). Inclusion of these features provided a deeper interpretability of the findings, with respect to raw data such as plasma concentrations of glucose, insulin, and C-peptide measured during specific metabolic tests (e.g., IM-IVGTT).

The application of machine-learning techniques to such a kind of database is the main novelty of this study in the context of T2DM risk assessment in women with a history of GDM. In fact, previous studies in the same context have focused on lipidomics or metabolomics (Lappas et al., 2015; Allalou et al., 2016; Khan et al., 2019). In particular, Lappas et al. (2015) aimed to determine whether circulating lipid levels 12 weeks following a pregnancy with GDM were associated with an increased risk of developing T2DM and identified lipid species CE 20:4, PE (P-36:2), and PS 38:4 as significant risk factors. Allalou et al. (2016) and Khan et al. (2019) proposed different predictive signatures, including different metabolites; moreover, reduced sphingolipids have been associated with the pathophysiology of transition from GDM to T2DM (Khan et al., 2019). Since “omics” approaches are still not typically viable in the clinical practice, our study proposed an easier alternative from the technical point of view. In addition, it should be noted that metabolomics/lipidomics predictive power could be enhanced complementing it with classical clinical and biochemical markers (Pallares-Méndez et al., 2016); therefore, our approach could be also used to complement, rather than replace, omics approaches when they will be available in clinical practice.

The main result of this study is the identification of DI, BSR of insulin, and mean area under the C-peptide concentration curve among the most relevant features for the progression to T2DM. DI represents the combined contribution of insulin secretion and insulin sensitivity and was already found to predict conversion to T2DM in a large epidemiological study (Lorenzo et al., 2010); BSR of insulin and mean area under the C-peptide concentration curve are indexes that provide a quantification of insulin secretion. Besides these indexes quantifying specific metabolic processes, age, BMI, and fasting glycemia have been selected.

Three different classification algorithms were tested, namely the decision tree, the Naïve Bayes, and the penalized logistic regression. Comparisons between models were based on AUC, CA, precision, sensitivity, specificity, and F1 and showed that logistic regression resulted the best model for the classification of progression to T2DM since it reported higher values than Naïve Bayes and decision tree in four out of six measures, specifically in AUC, CA, sensitivity, and F1. Naïve Bayes performed better only in precision and specificity, while decision tree reported lower values than the other models in all the measures. Moreover, logistic regression presented higher value of correct classification and lower value of misclassification, followed by decision tree and Naïve Bayes. Moreover, regularized regression methods (L1 or L2, as in this study) are characterized by including a small bias into the maximum likelihood estimation; the inclusion of this bias helps to reduce the variance, thus improving the predictions for new subjects (or the generalization of results) (Hastie, 2017).

Machine-learning techniques have been extensively explored in recent years for the prevention and management of T2DM (Huang et al., 2007; Yu et al., 2010; Perveen et al., 2016; Dalakleidi et al., 2017; Maniruzzaman et al., 2017; Zheng and Zhang, 2017; Talaei-Khoei and Wilson, 2018; Bernardini et al., 2019, 2020) but also showing possible criticalities. In fact, very often, the analysis with these techniques on large amounts of heterogeneous data leads to identify spurious correlations (Rumbold et al., 2020), indicating that the creation of appropriate databases, with selected groups of subjects and characteristics, as done in this study, is an aspect of primary importance and which cannot be disregarded in order to achieve reliable results. Thus, even though the considered population of women with a history of GDM was constituted by a limited number of subjects, they have been strictly controlled and monitored during a long follow-up. Moreover, the subjects have been carefully screened to detect outliers, and these were dropped out from the data set before the analysis; decision of dropping out the outliers instead of performing data imputation only for specific features was taken to reduce as much as possible bias and uncertainty. Adoption of machine-learning techniques, usually devoted to the analysis of large amounts of data, is justified in this study by the high number of features included in the generated data set, from which such techniques may allow determination of the most relevant ones. At the same time, when considering a low number of subjects with a high number of features, overfitting may occur and achieved results may be a bit optimistic, especially when using decision tree algorithm. It has to be acknowledged that this may be the risk of this study. However, well-known strategies have been adopted to mitigate this risk, namely, pruning (through which the redundant branches can be cut beforehand) and k-fold cross-validation (Zhou et al., 2021).

In this study, data analysis has been performed by using Orange (Demšar et al., 2013), an open-source data visualization, machine learning, and data mining toolkit. This software has the advantage of providing a visual programming front-end for explorative rapid qualitative data analysis and interactive data visualization; on the other side, possibilities in data analysis are limited by the procedures implemented in the Orange “building blocks.” Further studies may explore different and more customizable learning algorithms starting from the results of this study.

## Conclusion

This study was the first to apply machine-learning techniques to databases that contain features quantifying metabolic processes based on such standard clinical test in women with a history of GDM and at risk of developing T2DM. We found that DI, BSR of insulin, mean area under the C-peptide concentration curve, age, BMI, and fasting glycemia were identified as the most relevant features for the progression from GDM to T2DM. The obtained information from this pattern could be of interest for the study and characterization of diabetes pathophysiology.

## Data Availability Statement

The data analyzed in this study is subject to the following licenses/restrictions: The datasets generated and/or analyzed in the current study are available from AK-W on reasonable request. Requests to access these datasets should be directed to AK-W, alexandra.kautzky-willer@meduniwien.ac.at.

## Ethics Statement

The studies involving human participants were reviewed and approved by the Ethics Committee of the Medical University of Vienna. The patients/participants provided their written informed consent to participate in this study.

## Author Contributions

LI, AP, AT, and MM contributed to conception and design of the study. CG and AK-W contributed to acquisition of data. LI, AP, CG, LB, AK-W, AT, and MM analyzed and interpreted the data. LI, AT, and MM drafted the article. AP, CG, LB, and AK-W revised the article. All authors have read and approved the submitted version of the article.

## Conflict of Interest

The authors declare that the research was conducted in the absence of any commercial or financial relationships that could be construed as a potential conflict of interest.

## Publisher’s Note

All claims expressed in this article are solely those of the authors and do not necessarily represent those of their affiliated organizations, or those of the publisher, the editors and the reviewers. Any product that may be evaluated in this article, or claim that may be made by its manufacturer, is not guaranteed or endorsed by the publisher.

## Footnotes

1
https://orange.biolab.si/
